# High-Dose Vitamin C in Advanced-Stage Cancer Patients

**DOI:** 10.3390/nu13030735

**Published:** 2021-02-26

**Authors:** Anna Zasowska-Nowak, Piotr Jan Nowak, Aleksandra Ciałkowska-Rysz

**Affiliations:** 1 Department of Palliative Medicine, Chair of Oncology, Medical University of Lodz, Ul. Zeromskiego 113, 90-549 Lodz, Poland; aleksandra.cialkowska-rysz@umed.lodz.pl; 2 Department of Nephrology, Hypertension and Kidney Transplantation, Medical University of Lodz, Ul. Pomorska 251, 92-213 Lodz, Poland; piotr.nowak@umed.lodz.pl

**Keywords:** vitamin C, cancer, quality of life, pain, cancer-related fatigue, palliative care

## Abstract

High-dose intravenously administered vitamin C (IVC) is widely used in cancer patients by complementary and alternative medicine practitioners. The most frequent indications for IVC therapy result from the belief in its effectiveness as a potent anti-cancer agent which additionally enhances chemosensitivity of cancer cells and reduces chemotherapy-related toxicities and fatigue intensity. In this narrative review, we decided to deal with this issue, trying to answer the question whether there is any scientific evidence supporting the rationale for application of high-dose IVC therapy in advanced-stage cancer patients. Although results obtained from preclinical studies demonstrated that millimolar ascorbate plasma concentrations achievable only after IVC administration were cytotoxic to fast-growing malignant cells and inhibited tumor growth as well as prolonged the survival of laboratory animals, such positive effects were not found in human studies with advanced-stage cancer patients. We also have not found the rationale for the use of IVC to increase the effectiveness of chemotherapy and to reduce the chemotherapy-induced toxicity in the above mentioned group. Nevertheless, in palliative care, high-dose IVC might be considered as a therapy improving the quality of life and reducing cancer-related symptoms, such as fatigue and bone pain. However, because of the absence of placebo-controlled randomized trials on IVC efficacy in advanced-stage cancer patients, the placebo effect cannot be excluded.

## 1. Introduction

The discussion about the anti-cancer properties of vitamin C and its possible application in palliative care has been continued for over 50 years. Since it was proved that the route of vitamin C administration could affect its bioavailability and influence its potential anti-cancer properties [1], researchers, physicians, and cancer patients have become more interested in the method of the intravenous vitamin C (IVC) administration. Results obtained from in vitro studies revealed that millimolar ascorbate plasma concentrations, achievable only after intravenous vitamin C administration, are cytotoxic to fast-growing malignant cells. These results were also consistent with the data obtained from animal studies, where injections of high-dose vitamin C inhibited tumor growth and prolonged the survival of laboratory animals. The wide IVC popularity in the 2000s is evidenced by the data from the survey conducted by Padayatty et al. [2], in which an unexpectedly common use of vitamin C was reported among complementary and alternative medicine practitioners. Of 199 survey responders, 172 administered IVC to 11,233 patients in 2006 and to 8876 patients in 2008 with an average dose of 28 g every four days and 22 total treatments per patient, most frequently due to infection, cancer, and fatigue [2]. Although the scale of the use of high-dose IVC treatment in recent times is unknown, it seems to enjoy unflagging interest from patients with advanced-stage cancer disease, refractory to standard therapy. The most frequent questions asked by patients and their families to palliative medicine specialists concern the effectiveness of high-dose IVC as anti-cancer therapy and its use as an agent enhancing chemosensitivity of cancer cells or reducing chemotherapy-related toxicities and fatigue. In this narrative review, we decided to deal with this issue, trying to answer the question whether there is any scientific evidence supporting the rationale for application of high-dose IVC therapy in advanced-stage cancer patients.

## 2. Over 50 Years of History of the Anti-Cancer Properties of Vitamin C

Seventy years ago, McCormick noticed that vitamin C might be able to protect against cancer by increasing synthesis of collagen [3,4]. In the early 1970s, Cameron and Pauling [5] published the thesis claiming that ascorbic acid is able to potentiate the intrinsic production of serum physiological hyaluronidase inhibitor (PIH), thereby protecting against the spreading of cancer cells. Although reasons for hypotheses mentioned above were not clear, they have started out a widespread discussion about the value of vitamin C in cancer prophylaxis, supportive therapy, and palliative treatment [6]. In a later publication, Cameron and Pauling proved that ascorbic acid is directly involved in specific biochemical reactions that collectively support the natural host resistance to malignant invasive growth, through potentiation of stromal fibrosis, increased effectiveness of malignant cell encapsulation in fibrous tissue, extended lymphocytic infiltration, and protection of adreno-pituitary axis against depletion generated by cancer-related stress [6]. In the observational study conducted by Cameron and Campbell in 1974 [7], a daily dose of 10 g ascorbic acid was administered intravenously for 10 days followed by the same dose given orally to 50 terminal cancer patients. They observed various effects after ascorbate treatment. Tumor growth retardation and inhibition were noticed in 11 (22%) and three patients (6%), respectively, while no clinical response was observed in 17 patients (35%) and only minimal response was found in 10 patients (20%). On the other hand, in five patients (10%), tumor progression was noticed, but in other four patients (8%), a hemorrhage to a tumor occurred followed by its necrosis and accelerated patient’s death [7]. According to the authors, certain study participants also benefited from vitamin C therapy, demonstrating relief from bone pain due to metastases and from other mass effects (headache, Horner’s syndrome, laryngeal nerve paresis). Moreover, they revealed a significant reduction in the rate of re-accumulation of malignant ascites and malignant pleural effusion, reduction in the degree of hematuria, reversal of terminal malignant jaundice, or a decrease in the erythrocyte sedimentation rate [7]. In a subsequent publication, Cameron and Pauling [8] presented results of an observational case-control study in which 100 terminal cancer patients were given supplemental ascorbate as a part of their routine management and were compared to 1000 controls matched for sex, age, type of the primary tumor, and treatment. The study showed that the mean survival time was more than 4 times longer for the ascorbate treated subjects (receiving 10 g intravenously for 10 days and orally thereafter) than for controls not receiving vitamin C; moreover, survival time of 10% of the former group was 20 times greater than in controls [8]. Almost a twofold prolongation of average survival time in terminal cancer patients after vitamin C administration was observed also by Murata et al. [9].

In response to the data obtained by Cameron and Pauling, Creagan et al. [10] conducted in 1979 a randomized, controlled double-blind trial to evaluate the effect of vitamin C (10 g daily by oral route) on the severity of symptoms and survival rate in 123 patients with advanced and preterminal cancer. The study proved a lack of vitamin C effect. There were no statistically significant differences in survival time between ascorbate and control groups [10]. Although study participants reported improved appetite, strength, activity level, and pain control, there were also no statistically significant differences in the severity of symptoms between two treated groups [10]. Similarly, Moertel et al. [11] in a double-blind placebo-controlled study with 100 advanced colorectal cancer patients randomly assigned to treatment either with 10 g of vitamin C daily or placebo showed no advantage with regard to either the interval between the beginning of treatment and disease progression or patient survival time. Based on subsequent experiments, it is now known that results obtained by Creagan et al. and Moertel et al. should not be compared with those of Cameron and Pauling because of a different route of vitamin C administration [12]. We discussed this issue in the next chapter.

About twenty years later, Padayatty et al. [13], basing on the evidence that millimolar vitamin C plasma concentrations achieved only through intravenous (but not oral) administration can be toxic to some cancer cells [14,15,16,17,18], proposed that ascorbate treatment of cancer should be re-examined. They published three well-documented cases of advanced cancers where patients had unexpectedly long survival times after receiving high-dose intravenous vitamin C (15–65 g twice a week for 2–10 months, followed by continuation in smaller doses for 1–4 years) as their only significant therapy [19]. Since then, numerous studies have investigated the effects of high-dose vitamin C on tumor growth, survival, impact on quality of life (QoL), side effects associated with chemotherapy and radiation therapy, cancer-related symptoms, and interactions with conventional anti-cancer therapy [20].

## 3. Vitamin C Intravenous Versus Oral Route

In the studies of Cameron et al. [7,8,21,22], ascorbic acid given intravenously in daily doses of 10 g was recognized as effective in the treatment of cancer. This effect was not observed in double-blind placebo-controlled trials conducted in the Mayo Clinic, when the same doses of ascorbate were given only by the oral route [10,11], leading to the conclusion that the route of administration may result in differences of ascorbate bioavailability which influences its potential anti-cancer properties.

When given orally, ascorbate concentration in plasma of healthy humans is strictly controlled by at least three mechanisms, namely intestinal absorption (bioavailability), tissue accumulation, and renal elimination [23]. The rate of ascorbic acid utilization is the additional mechanism that may be meaningful in disease states [23]. For this reason, ascorbate concentration in plasma does not exceed 100 μmol/L when it is supplied orally with food; even with oral supplementation approaching maximum tolerated doses, it is always <250 μmol/L [23]. It was demonstrated in a depletion-repletion pharmacokinetic study conducted in healthy volunteers that, with the oral daily dose of 100 mg, the concentration of ascorbate in daily fasting plasma reaches a plateau between 50–60 µmol/L [24]. Whereas increasing the daily dose ten times to 1000 mg gives only a slight increase in plasma concentration to 70–85 μmol/L, with a subsequent minimal additional increase resulting from the dose of 2500 mg (plasma concentrations about 80–90 μmol/L) [24]. Intracellular ascorbate concentrations in circulating immune cells (neutrophils, monocytes, and lymphocytes) reached maximum at the 100 mg daily dose of vitamin C and did not increase at higher doses [24]. Bioavailability is 100% after a single dose of 200 mg, and it declines at higher vitamin C doses, reaching about 33% after a single dose of 1250 mg [24].

By contrast, after intravenous infusion, the plasma concentration of ascorbate achieves higher values than after the administration of the same oral doses and the difference increases with the dose [1]. The intravenous route bypasses the regulation of intestinal uptake of oral vitamin C dependent on the sodium-dependent transporter-1 (SVCT1), resulting in an uncontrolled rise in plasma concentration [25]. After intravenous administration of 1.25 g of ascorbic acid in healthy volunteers, the peak plasma ascorbate was about 6.5-fold greater than after the administration of the same dose given by the oral route [1]. In the same study, pharmacokinetic modeling predicted the plasma ascorbate concentration of 220 μmol/L for the maximum tolerated oral dose of 3 g every 4 h, compared to 13,350 μmol/L (13.35 mmol/L) and 15,380 μmol/L (15.38 mmol/L) for 50 g and 100 g intravenous single doses, respectively [1]. It is worth mentioning that intravenous administration can lead to even 30 to 70-fold higher plasma levels of ascorbate than the highest tolerated oral dose [1]. However, these high concentrations are relatively transient due to the rapid clearance by the kidneys resulting in a half-life of about 2 h in circulation [25]. In cancer patients either undergoing or not receiving chemotherapy, intravenous administration of vitamin C in a single dose of 0.1–3.0 g/kg of body weight resulted in maximum plasma concentrations C_max_ ranging from 2 to 37 mmol/L [25,26,27,28,29,30,31,32].

## 4. Biological Properties of Vitamin C

Vitamin C (L-ascorbic acid) is a water-soluble vitamin with a variety of biologically important functions. Humans and few other mammals do not possess the capacity of vitamin C biosynthesis from glucose due to the lack of the enzyme L-gulono-γ-lactone oxidase, essential for this metabolic pathway [33]. Most vitamin C biological functions are associated with the presence of a lactone ring with electron rich 2-en-2,3-diol-1-one moiety in its molecule [34]. Under physiological pH conditions, vitamin C exists predominantly as the ascorbate anion, arising from the completely dissociated 3-hydroxyl group [34]. Although ascorbic acid serves both as a reducing agent and antioxidant, it is best known for its capacity to act as an antioxidant able to undergo two consecutive one-electron oxidation reactions and deprotonation of both hydroxyl groups at positions 2 and 3, resulting in the formation of dehydroascorbic acid (DHA), which is unstable in neutral pH [34,35]. In the above mentioned process, intermediate radical species are formed, such as relatively stable ascorbyl radical (Asc^●^) [35,36]. Although dehydroascorbic acid and ascorbate free radicals can be easily reduced by both NADH-and NADPH-dependent mechanisms, the main pathway of ascorbate recycling is enzymatic, mediated largely by glutaredoxin and thioredoxin reductase [37].

Ascorbate is transported to the tissues through the bloodstream and actively taken up by most of the cell types through sodium-dependent vitamin C transporters SVCT-1 and SVCT-2, reaching intracellular concentrations several folds higher than plasma levels (about 50 µmol/L) [34,35]. Red blood cells do not express SVCT 2 and accumulate ascorbate by the uptake of dehydroascorbic acid through sodium-independent facilitative GLUT type glucose transporters, followed by intracellular reduction to ascorbate [34]. In the plasma of healthy humans, the reduced form of ascorbate is dominant, while dehydroascorbic acid is present at a very low level, in the range of 0–2 µM or about 1–2% of ascorbate concentrations [34,37]. In some organs, such as brain, adrenals, liver, and white blood cells, high intracellular ascorbate concentrations are found, ranging up to 20 mmol/L, in response to the demand for ascorbate as an essential enzyme cofactor [35].

Ascorbate is a potent antioxidant due to the ability to donate electrons. It scavenges reactive oxygen species (ROS) and reactive nitrogen species (RNS), providing effective antioxidant protection against free-radical damage to many tissues. Studies demonstrate that ascorbate markedly improved the endothelium-dependent response to degenerative processes caused by oxidant stress [38,39], reduced low-density lipoprotein peroxidation in plasma [40,41,42,43,44], modulated the monocyte intercellular adhesion molecule 1 (ICAM-1) gene expression [45], and reduced oxidative stress in brain [46]. It also decreased the steady-state level of oxidative DNA damage to mononuclear blood cells [47] and protected neutrophils against ROS generated during phagocytosis [33]. Moreover, ascorbate participates in the regeneration of other antioxidants such as glutathione and α-tocopherol back to their active state [33]. In animal studies, the administration of vitamin C, due to its antioxidant effect, was able to ameliorate the toxic effect of methotrexate in liver and kidney tissues, which occurred because of enhanced oxidative stress [48].

On the other hand, vitamin C exhibits a potent pro-oxidant activity, mainly due to very good reducing properties of ascorbate, which is able to reduce catalytic metals such as Fe^3+^ to Fe^2+^ and Cu^2+^ to Cu^+^, increasing the pro-oxidant chemistry of these metals and facilitating the generation of reactive oxygen species [34]. Reactions of ascorbate with oxygen or with free transition metal ions lead to the generation of superoxide, H_2_O_2_ and highly reactive oxidants, such as the hydroxyl radical by promoting the Fenton chemistry [35]. The ability to reduce transition metals is regarded as fundamental to the activity of ascorbate as an essential co-factor for reactions requiring reduced iron or copper metalloenzymes [33,35].

Ascorbate participates in the stabilization of collagen tertiary structure, carnitine biosynthesis, and biosynthesis of some hormones (corticosteroids, aldosterone) as well as amino acids [33]. It influences collagen gene expression, cellular procollagen secretion, and the biosynthesis of other connective tissue components including elastin, fibronectin, proteoglycans, bone matrix, and elastin-associated fibrillin [33]. Moreover, it is necessary for the biosynthesis of catecholamines (norepinephrine and epinephrine) and is involved in tyrosine metabolism as well [33]. Ascorbate regulates the transcription of hypoxia inducible factor-1α (HIF-1α) [25] and acts as a co-factor for enzymes named ten-eleven translocation (TET) family dioxygenases, essential in the demethylation of 5-methylcytosine in DNA [49]. The Jumonji C (JmjC)-domain-containing histone demethylases also require ascorbate as a co-factor for histone demethylation [50]. Moreover, ascorbic acid increases the intestinal absorption of non-heme iron from the diet and participates in the maximization of some hormones activity, such as cholecystokinin, oxytocin, vasopressin, and alpha-melanotropin [51]. Anti-inflammatory properties of ascorbic acid result in the reduction of cytokines, chemokines, and C-reactive protein (CRP) levels in plasma [52,53].

## 5. Vitamin C Plasma Concentrations in End-Stage Cancer Patients

Several large epidemiological studies conducted in Europe and North America revealed that from a few to even 50% of healthy adults can have hypovitaminosis C, defined as the plasma concentration less than 23 μmol/L, despite the use of diet containing recommended daily doses of vitamin C [54]. Severe vitamin C deficiency (plasma concentration <11 μmol/L) can be a problem concerning 1.4% to 20% of healthy adults, making them more likely to develop clinical manifestations of scurvy [55]. That percentage can be significantly higher in cancer patients [26,56,57,58,59,60,61,62,63,64,65] mainly due to the enhanced oxidative stress and inflammatory processes that increase utilization of ascorbate. Furthermore, impaired oral intake or a history of surgery or radiation reducing absorptive surfaces [51] makes the compensation for the increased demand for ascorbic acid impossible. What is more, the need for vitamin C increases during anti-cancer therapy [59,63,66,67,68,69] and treatment of related complications [70,71,72]. In a Danish study among 20 patients with myeloid cancers who were undergoing treatment with 5-azacytidine at baseline, 14 patients were vitamin C deficient and in eight cases, the deficiency was profound [59]. Moreover, as the disease stage progresses, ascorbate plasma levels decline in comparison with an early cancer stage and with healthy subjects as well [51,57]. In a study by Mayland et al. [56], severe vitamin C deficiency was found in 30% of hospice residents with advanced cancer. Riordan et al. [58] observed severe ascorbate deficiency in 50% of terminal cancer patients. It was observed that the low dietary intake, low albumin plasma concentration, high platelet count, high CRP level, disseminated cancer, and shorter survival were significantly associated with low plasma vitamin C in the above mentioned group [56].

Symptoms of vitamin C deficiency include fatigue, weakness, poor wound healing, ecchymoses, xerosis, lower extremity edema, and musculoskeletal pain [51]—most of them are often observed in end-stage cancer patients. The Recommended Dietary Allowance (RDA) for vitamin C is 90 mg/day for adult men and 75 mg/day for adult women, however it is proposed that recommended vitamin C intakes should be based on body weight or optimum plasma concentration about 70 μmol/L [54,73].

## 6. Potential Anticancer Properties of Vitamin C

Potential anticancer properties of ascorbic acid have been known since the 1960s, when it was shown to be highly toxic or lethal to Ehrlich ascites carcinoma cells in vitro, while it remained nontoxic to normal body tissues [74]. The first hypotheses emphasized highly reactive oxidation-reduction properties of the ascorbic acid-dehydroascorbic acid system that might be capable of interfering with some metabolic pathways characteristic for cancer cells, such as anerobiosis known as the Warburg effect and differences in electrical charge on their membranes compared to normal cells [6,75,76]. Benade et al. [74] in 1969 proposed that the main cytotoxic mechanism of ascorbate can be connected with intracellular generation of hydrogen peroxide (H_2_O_2_) produced upon oxidation of vitamin C. This occurs because cancer cells selectively take up more ascorbate compared to normal cells through the facilitated transport with participation of glucose transporters (GLUTs) due to an increased metabolic need for glucose [20]. In the in vitro study, it was demonstrated that ascorbate cytotoxicity towards cancer cells is increased synergistically by the concomitant administration of 3-amino-1,2,4-triazole (ATA) which specifically inhibits catalase activity in cancer cells (catalase catalyzes decomposition of hydrogen peroxide H_2_O_2_ to oxygen and water), thereby decreasing the ability of cancer cells to detoxify the H_2_O_2_ effectively. Additionally, it was found that catalase activity in cancer cells is 10–100-fold lower than in normal cells, making them over-sensitive to ascorbate [74]. The next studies conducted in vitro on cancer cells revealed that both ascorbate and its oxidation product, dehydroascorbate, at high doses were cytotoxic or lethal to fast-growing malignant cells [14,77], but less toxic to non-malignant cells [14]. In addition, xenograft experiments showed that parenteral ascorbate in the dose of 4 g/kg body weight, administered as the only treatment once or twice daily and leading to an increased blood and tissue ascorbate concentration >30 mmol/L, significantly (*p* < 0.05) decreased both tumor growth and weight rates of glioblastoma, ovarian and pancreatic carcinoma cells in mice [78]. Other animal studies indicated that parenterally administered high doses of vitamin C (0.1–8 g/kg body weight daily for 2–4 weeks) not only inhibited tumor growth but also reduced invasiveness of cancer cells and increased survival [25,78,79,80,81,82,83,84,85].

To determine whether ascorbate at physiological (0.1 mmol/L) and pharmacological (0.3–20 mmol/L) concentrations is able to selectively kill cancer cells and to establish its mechanism, Chen et al. [78,86] incubated normal and cancer cell types in cell culture media containing appropriate ascorbate concentrations ranging from 0 to 20 mmol/L. It was shown that normal cells were unaffected by 1–2 h incubation in 20 mmol/L ascorbate, whereas 5 cancer cell lines had EC_50_ values of <4 mmol/L [86], and EC_50_ values were <10 mmol/L for other 75% tumor cells tested [78]. Cancer cell death observed by Chen et al. [86] was associated with extracellular, not intracellular ascorbate. Moreover, it was dependent on extracellular H_2_O_2_ formation upon oxidation of ascorbate and displayed a linear relationship with ascorbyl radical (Asc^●^) formation as an intermediate. Ascorbate generated detectable levels of H_2_O_2_ in extracellular medium in the presence of serum, but not in whole blood, indicating that ascorbate at high concentrations achieved only during intravenous administration may be a pro-drug for H_2_O_2_ delivery to tissues [86]. In a study by Maramag et al. [17], the treatment of human prostate cancer cells with vitamin C resulted in a dose- and time-dependent decrease in cell viability and thymidine incorporation into DNA. Addition of catalase, an enzyme that degrades hydrogen peroxide to culture medium inhibited the above mentioned effects, whereas superoxide dismutase, an enzyme that dismutates superoxide and generates hydrogen peroxide, did not prevent decrease in cell numbers and DNA synthesis, supporting the thesis that the cytotoxic effect of vitamin C is related to the production of hydrogen peroxide [17]. Makino et al. [16] and Sakagami et al. [18] observed that induction of cell death in human renal carcinoma, glioblastoma, and promyelocytic leukemic HL-60 cell lines was caused by ascorbic acid derivatives which produce ascorbyl radicals during the oxidative degradation (sodium-L-ascorbate, L-ascorbic acid, D-isoascorbic acid, sodium 5,6-benzylidene-L-ascorbate, and sodium-6-beta-O-galactosyl-L-ascorbate). Whereas L-ascorbic acid 2-phosphate magnesium and L-ascorbic acid 2-sulfate dipotassium salt, which do not produce the ascorbyl radical, were inactive, suggesting the possible role of the ascorbyl radical in cell death induction [16]. A catalytic concentration of copper increased ascorbate toxicity to melanoma cells two to five-fold, whereas cytotoxicity to other cells increased only 1 to 1.5 times [15]. Moreover, exposure of HL-60 cells and human bladder tumor cells to ascorbic acid or its active derivatives resulted in the rapid elevation of intracellular Ca^2+^ concentration, which might serve as the initial signal leading to the cell death pathway [18,87].

Based on the data from the above-mentioned experiments, it has been proposed that ascorbate-related cancer cell death was mediated by H_2_O_2_ formation, which in the presence of reduced transition metal catalysts is able to produce the highly reactive hydroxyl radical (OH^●^) species [23]. In the extracellular fluid, a molecule of ascorbic acid loses one electron and forms an ascorbate radical, whereas this electron subsequently reduces ferric iron to ferrous iron, as it was shown in the chemical reaction presented below [23]:

AscH^−^ + Fe^3+^ → Fe^2+^ + Asc^●−^ + H^+^

The above mentioned complex donates an electron to molecular oxygen, forming a superoxide anion (O2^●−^) reacting with hydrogen to produce H_2_O_2_:

2O_2_ → 2O_2_^●−^ + 2H^+^ → H_2_O_2_ + O_2_

Then, in the classic Fenton reaction, ferrous iron is oxidized by H_2_O_2_ to generate ferric iron and a highly reactive hydroxyl radical (OH^●^) [23]:

Fe^2+^ + H_2_O_2_ → Fe^3+^ + OH^●^ + OH^−^

It is also likely that reduced metal centers (Fe^2^) can donate their electron to molecular oxygen to produce superoxide radical anions (O_2_^●^^−^) [23]:

Fe^2+^ + O_2_ → Fe^3+^ + O_2_^●−^

Ascorbate as a good pro-oxidant converts Fe^3+^ back to Fe^2+^ to continue the cyclic oxidation/reduction of Fe^2+^ coupled to the generation of superoxide radical anions [35]. A possible mechanism of cell killing by high intracellular concentrations of ascorbate can involve the mobilization of intracellular iron storage by ascorbate, followed by the Fenton reaction to give highly reactive hydroxyl radicals [88].

The presented hypothesis was confirmed in the studies conducted in rodents [78,89]. In mice bearing glioblastoma xenografts, it was shown that a single pharmacologic dose of vitamin C produced a sustained ascorbate radical and hydrogen peroxide formation selectively within interstitial fluids of the tumor tissue (but not in blood) [78]. Moreover, after parenteral vitamin C administration, Asc^●−^ achieved blood concentrations <5 nmol/L (even when corresponding ascorbate concentrations were >8 mmol/L), while Asc^●−^ concentrations in the extracellular fluid of the tumor tissue were from 4 to 12-fold higher than those achieved in the blood and were a function of ascorbate concentrations [89]. However, the studies conducted so far have not answered the question why ascorbate throughout the production of H_2_O_2_ is able to kill some cancer cells while it is nontoxic to normal cells. It was demonstrated that H_2_O_2_ is generated in the extracellular space, but because H_2_O_2_ is a membrane-permeable agent, the targets for its action can be located extra- and intracellularly as well, generating oxidative cell damage [35,86]. It is suggested that extracellular H_2_O_2_ might target membrane lipids, forming hydroperoxides or reactive intermediates that are repaired in normal but not cancer cells. Moreover, intracellular H_2_O_2_ could target deoxyribonucleic acid (DNA) or DNA repair proteins [86]. Superoxide radical anions and H_2_O_2_ are important signaling molecules involved in pro-inflammatory responses among other pathways [90].

As it was mentioned above, both ascorbic acid and dehydroascorbic acid added to culture media at high doses were cytotoxic to cancer cells. It is noteworthy that in cell culture media, vitamin C is oxidized to DHA with a half-life about 70 min, unless reducing agents are added [91]. To answer the question about the mechanism by which DHA is able to kill cancer cells while sparing normal cells, Yun et al. [91] conducted the study reporting that cultured human colorectal cancer cells with KRAS (Kirsten rat sarcoma viral oncogene) or BRAF (v-raf murine sarcoma viral oncogene homolog B) mutations were selectively killed when exposed to high concentrations of vitamin C, and that effect resulted from an increased DHA uptake via the GLUT1 glucose transporter. Inside the cell, DHA is reduced by GSH, NADH, and NADPH-dependent enzymes leading to the depletion of glutathione, thioredoxin, and NADPH, thus increasing the intracellular oxidative stress [35]. ROS accumulation inside cells inactivates glyceraldehyde 3-phosphate dehydrogenase (GAPDH), which leads to a decreased formation of glycolytic adenosine 5’-triphosphate (ATP) and pyruvate, causing an energetic crisis that triggers cell death [91]. Such cytotoxic effect was subsequently confirmed in *Apc/Kras^G12D^* mutant mouse intestinal cancers [91]. As KRAS and BRAF mutations are found in approximately 40% and 10% of human colorectal cancers (CRCs), respectively, the therapeutic use of vitamin C in treating patients with colorectal cancer presenting such type of mutations seems to be rational [91].

It has been also proved that ascorbate at physiological concentrations significantly suppressed hypoxia-inducible factor 1α (HIF-1α) protein levels in cancer cell lines [92,93,94]. Ascorbate as a cofactor for iron-dependent HIF-hydroxylase enzymes keeps the iron enzyme in the active state (Fe^2+^) and is involved in controlling both protein stability and the formation of an active transcription complex modulating the cell survival response [95]. In a state of ascorbate deficiency, HIF-hydroxylase activity is compromised and HIF transcription activity is increased, especially in the environment of mild or moderate hypoxia which is characteristic for neoplastic tissue [35]. On the other hand, it is proposed that increasing ascorbate supplied to cancer cells could stimulate the activity of the HIF-hydroxylases and slow tumor growth rates by the suppressed activation of the HIFs [35].

Ascorbate is also a cofactor for ten-eleven translocation family dioxygenase (TET) enzymes, involved in the hydroxylation of 5-methylcytosine (5mC) into 5-hydroxymethylcytosine (5hmC), and further to 5-formylcytosine (5fC) and to 5-carboxylcytosin (5caC), thus modulating DNA methylation [49,50]. The loss of 5hmC has recently been identified as a novel hallmark for some types of cancer, such as malignant melanoma [96]. Studies have shown that overexpressing TET2 in melanoma cells and TET1 in breast cancer cells increase their 5hmC content and decrease their malignancy in vitro and in vivo [96]. Addition of ascorbate into the medium in a dose- and time-dependent manner promotes TET activity, leading to a rapid and enhanced generation of 5hmC in cultured cell lines [97,98]. It has been also proved by Gustafson et al. [96] that vitamin C at both physiological and pharmacological concentrations is able to re-establish the 5hmC global content in melanoma cells towards that in normal melanocytes, while decreasing its invasiveness and clonogenic growth.

In experimental rodents after parenteral administration of vitamin C, not only the increased production of H_2_O_2_ was observed [83], but also the altered expression of genes involved in protein synthesis, cell cycle progression and angiogenesis [82,91,99], reduced levels of HIF-1 and vascular endothelial growth factor protein VEGF [100,101]. Moreover, vitamin C has been shown to generate the cell cycle arrest (G0/G1), caspase-independent autophagy mediated by beclin-1 and LC3 II, apoptosis via induction of apoptosis-inducing factor (AIF), induction of oxidative DNA damage, apoptosis mediated by Bax protein signalling, release of cytochrome C from mitochondria, activation of caspase 9 and caspase 3, and cleavage of poly[ADP-ribose] polymerase in a ROS-dependent manner [20].

Although anti-cancer properties of vitamin C still remain not fully explained, there have been numerous human studies conducted so far to evaluate its effectiveness in cancer therapy. Some of them seem to be promising especially in the context of positive effects on quality of life, symptoms, and chemotherapy-related adverse effects [19,26,28,29,30,31,32,102,103,104,105,106,107]. In the further discussion on the benefits of high-dose IVC therapy in patients with advanced-stage cancer, we relied on the data from studies conducted among patients whose profile best suited patients under palliative care, namely patients with disseminated neoplastic disease, resistant to standard anti-cancer treatment.

## 7. Does High-Dose IVC in Monotherapy Is an Effective Anti-Cancer Agent in Patients with Advanced-Stage Malignancies?

Although the results obtained from animal models demonstrating a significant reduction in tumor growth were very promising, the administration of high-dose IVC as a single anti-cancer agent does not find support among the oncology specialists who conduct anti-cancer therapy. To evaluate the effectiveness of high-dose IVC therapy as the sole anti-cancer treatment in advanced stage of malignancy among clinical studies conducted so far, we identified four nonrandomized phase I or II studies with 88 participants [27,28,29,58] and one report presenting seven clinical cases [108]. All participants of analyzed studies had advanced malignancies with distant metastases or cancer disease refractory to standard anti-cancer therapy.

In a phase I clinical trial conducted by Stephenson et al. [28] to evaluate the safety, tolerability, and pharmacokinetics of high-dose IVC among 17 study participants with solid tumors refractory to standard therapy, a 4-week IVC treatment was administered in doses ranging from 30 to 110 g/m^2^ for 4 consecutive days in a week. None of the subjects experienced an objective tumor-related response (13 patients had progressive disease, three patients presented stable disease, one patient was withdrawn from the study) [28]. Similarly, Hoffer et al. [29] in a phase I clinical trial conducted in patients with advanced malignancies, administered IVC in doses ranging from 0.4 to 1.5 g/kg body weight three times weekly for an average 10 weeks and did not observe an objective tumor-related response in any of 24 study participants. Neither disease remission nor significant reduction in tumor growth was also reported by Nielsen et al. [27] in a study group of chemotherapy-naive patients with metastatic, castration-resistant prostate cancer receiving weekly infusions of IVC (5, 30, and 60 g a week in 1st, 2nd, and 3rd–12th week, respectively). Riordan et al. [58], among 24 late stage terminal cancer patients receiving continuous IVC infusion of 150–710 mg/kg/day for up to eight weeks, reported only one patient with stable disease who continued therapy for 48 weeks.

On the other hand, Riordan et al. [108] presented complete remission after sole IVC administration of 15–65 g twice a week in two patients with metastatic renal cell carcinoma and in one patient with non-Hodgkin’s lymphoma receiving IVC in monotherapy after the chemotherapy was stopped due to side effects. Authors also presented one case of resolution of skull metastases in a disseminated end-stage breast cancer patient receiving vitamin C in doses of 100 g three times a week and no recurrence in a woman with non-Hodgkin’s lymphoma (diffuse large cleaved cell of B-cell lineage) receiving IVC as the only treatment after completed radiotherapy [108].

The results of the clinical studies presented above clearly indicate no consistent evidence for anti-cancer efficacy of high-dose IVC therapy administered in patients with advanced stage of neoplastic disease, particularly in terms of objective tumor-related response or improved survival outcomes. Single cases of complete cancer remission or reduction in metastatic lesions cannot be reliable evidence for anti-cancer effectiveness of IVC monotherapy without the confirmation in high-quality clinical trials.

## 8. Does High-Dose IVC Increase the Effectiveness of Chemotherapy in Patients with Advanced-Stage Cancer?

It has been demonstrated in in vitro studies that ascorbic acid added to the medium enhanced the susceptibility of cancer cells to etoposide, cisplatin, and doxorubicin, and it can act synergistically with gemcitabine [35]. Moreover, animal studies have indicated that concomitant administration of vitamin C with many chemotherapeutic agents and radiotherapy works synergistically, resulting in a decreased tumor size and weight rates and increased survival as well [25]. To evaluate the effectiveness of high-dose IVC therapy as an agent enhancing chemosensitivity of cancer cells in the advanced stage of malignancy, we compiled the results of clinical trials conducted so far in this area. We analyzed only those studies, where study participants had advanced malignancies with distant metastases or refractory to standard anti-cancer therapy. By searching the database, we identified only one non-blinded randomized controlled trial meeting the above criteria (*n* = 25 ovarian stage III and IV cancer patients) [31], two nonrandomized phase I studies conducted among patients with disseminated pancreatic cancer (*n* = 25) [30,32], and three phase I or II trials conducted among patients with drug-resistant multiple myeloma (*n* = 68) [109,110,111,112].

Based on the observations that the combination of parenteral ascorbate with the conventional chemotherapeutic agents, carboplatin and paclitaxel, synergistically inhibited ovarian cancer in preclinical ovarian cancer models, Ma et al. [31] conducted a clinical trial assessing effectiveness and toxicity of such treatment in patients with stage III and IV ovarian cancer. Study participants were randomized into either the standard paclitaxel/carboplatin therapy administered for the initial six months (*n* = 12) or the paclitaxel/carboplatin therapy administered for six months with the concomitant ascorbate treatment for 12 months (*n* = 13). Although overall survival tended to be improved based on the Kaplan–Maier analysis, and the median time for disease progression was 8.75 months longer with ascorbate addition to standard chemotherapy than in chemotherapy alone, neither therapy achieved statistical significance [31].

In nonrandomized phase I studies, both Welsh et al. [30] and Monti et al. [32] observed some efficacy of IVC administration in combination with gemcitabine and gemcitabine/erlotinib in patients with metastatic pancreatic cancer (reduction in size of the primary tumor in imaging tests, improved performance status, median overall survival of 13 months and 182 days, respectively). However, these studies were carried out on a small sample size (nine subjects who completed the study in both trials), which does not allow for unambiguous statistical conclusions regarding the advantage of such management over the use of chemotherapy alone.

After the previous in vitro study demonstrating that ascorbic acid enhances the activity of arsenic trioxide (As_2_O_3_) against drug-resistant multiple myeloma (MM), Bahlis et al. [109] conducted a phase I clinical trial to evaluate effectiveness and toxicity of such combined therapy in the group of six patients with stage IIIA relapsed or refractory MM. It was observed that the co-administration of 1 g IVC following the As_2_O_3_ infusion five days a week for five consecutive weeks followed by two weeks of rest (patients were allowed to receive a maximum of six cycles) did not alter the pharmacokinetics of arsenic trioxide with acceptable toxicity. Two patients had partial responses and four patients presented a stable disease [109]. Similar observations were achieved by other researchers studying efficacy and safety of IVC administration in combination with standard treatment in patients with resistant MM. Abou-Jawde et al. [110] and Wu et al. [111], in a phase II clinical trial, conducted among groups of 20 patients receiving the combination of arsenic trioxide, dexamethasone, and intravenously administered 1 g ascorbic acid, observed the overall response rate in 30% and 6%, respectively, with median overall survival of 962 days and 11 months and progression-free survival of 316 days and 4 months, respectively. Berenson et al. [112] observed an objective response in 27% of 22 study participants receiving the combination of arsenic trioxide, bortezomib, and ascorbic acid with median progression free-survival of 5 months, 12-month progression-free survival, and overall survival rates of 34% and 74%, respectively.

Riordan et al. [108] in a clinical report presented one case of complete remission in a patient with stage IV colorectal carcinoma receiving combined chemotherapy (5-FU/Leucovorin) with 100 g IVC weekly and one case of no disease progression in a patient with metastatic low-grade mucinous carcinoma of pancreas undergoing chemotherapy in combination with 75 g IVC weekly.

The results of the clinical trials presented above indicate no reliable evidence for enhanced anti-cancer effectiveness of conventional therapy in combination with high-doses of IVC. Although the data from the presented clinical trials seems to be promising, reliable evidence for anti-cancer effectiveness of such a procedure is not possible to maintain without the confirmation in high-quality clinical trials with chemotherapy alone as a comparator.

## 9. Does High-Dose IVC Reduce the Chemotherapy-Induced Toxicity in Patients with Advanced-Stage Cancer?

To answer the question whether the high-dose IVC therapy administered concomitantly with standard chemotherapy reduces its cytotoxicity in patients with advanced cancer, we identified a total of seven studies including 118 patients: one non-blinded randomized controlled trial (*n* = 25 participants) [31] and six non-randomized phase I or II studies (*n* = 93) [30,32,109,110,111,112]. Study participants of analyzed studies had advanced malignancies with distant metastases or disease refractory to standard anti-cancer therapy.

Ma et al. [31] reported that the addition of ascorbic acid to standard paclitaxel/carboplatin therapy in stage III and IV ovarian cancer patients reduced chemotherapy-induced toxicity. IVC treatment did not increase the rate of graded 3 or 4 toxicities using the National Cancer Institute Common Terminology Criteria for Adverse Events version 3 (CTCAEv3) and there were also no observed grade 5 treatment-related toxicities (death). Moreover, the authors proved that grade 1 and grade 2 toxicities (such as neurotoxicity, bone marrow toxicity, infections, hepatobiliary/pancreatic toxicity, dermatological toxicity, and toxicities in the skin, renal, genitourinary, pulmonary, and gastrointestinal systems) were significantly decreased in the group receiving ascorbic acid with standard chemotherapy when compared to sole chemotherapy. Unfortunately, they did not show detailed data about all the categories of cytotoxicity evaluated [31].

In two phase I clinical trials, no evidence for increased toxicity of concurrent treatment with gemcitabine, erlotinib, and ascorbic acid was found in patients with advanced pancreatic cancer [30,32]. None of the adverse effects observed during these studies appeared to be specifically attributed to the ascorbic acid treatment since each of these adverse events is frequently recorded in the progression of pancreatic cancer patients and/or gemcitabine and erlotinib treatment [32]. Hematologic toxicities (leukopenia, lymphopenia, neutropenia, thrombocytopenia) were consistent with percentages reported for these toxicities induced by gemcitabine alone [30], while laboratory toxicities (elevated GGT, hypocalcemia) and serious adverse events, such as internal bleeding, ileus, and death, were likely related to the disease progression [30,32]. In addition, in the studies evaluating the safety of IVC administered simultaneously with the standard treatment of drug-resistant multiple myeloma (arsenic trioxide in monotherapy, arsenic trioxide/dexamethasone, and arsenic trioxide/bortezomib), therapy-related toxicities were related mainly to arsenic trioxide properties [109,110,111,112].

Because of a specific design of most of the above mentioned studies lacking appropriate comparators, a proper evaluation of effectiveness of high-dose IVC therapy in reduction of toxicities related to anti-cancer therapy is not possible. Data based on one non-blinded randomized controlled trial with 25 participants, even promising, is not sufficient to make clear conclusions about effectiveness of IVC therapy in the reduction of chemotherapy-induced toxicity in advanced-stage cancer patients.

## 10. Does High-Dose IVC Affect the Quality of Life in Advanced-Stage Cancer Patients?

According to the World Health Organization (WHO) definition, palliative care is an approach that improves the quality of life of patients and their families facing the burden of life-threatening illness through prevention and relief in suffering by early identification, adequate assessment and treatment of pain and other problems (physical, psychosocial, spiritual) as well [113].

Health-related quality of life (HRQoL) is defined as a subjective assessment of well-being perceived by an individual, including the ability to perform everyday activities, and patient’s satisfaction with the level of functioning and control of a disease [114]. It covers the subjective perception of the positive and negative aspects of cancer symptoms, physical, emotional, social, and cognitive functions and side effects of treatment [115]. QoL is usually seen as a composite of a life situation in relation to the culture in which individuals live, their system of values, goals, expectations, and interests [116].

The European Organization for Research and Treatment of Cancer QLQ-C30 (EORTC QLQ-C30) questionnaire is approved as the most commonly used instrument evaluating HRQoL in clinical trials in oncology. The EORTC QLQ-C30 is a 30-item questionnaire that consists of nine multi-item scales: five functional scales (physical, role, cognitive, emotional, and social), three symptom scales (fatigue, pain, nausea/vomiting), as well as global health and global quality-of-life scales [117]. The remaining part contains questions about additional symptoms commonly reported by cancer patients (dyspnea, appetite loss, sleep disturbance, constipation, and diarrhea) and the perceived financial impact of the disease and treatment [117]. The measures range in score from 0 to 100, with a difference of 4–10 points representing a small change, and a difference of 10–20 points representing a medium change in QOL [117].

Palliative care focused on improving the quality of life is of particular importance in advanced cancer disease refractory to anti-cancer therapy. However, epidemiological data indicates that even 82% of all cancer patients have low QoL scores, in the majority of them resulting from burdensome symptoms [118]. Among terminal cancer patients at hospices, the prevalence of patients with low QoL is about 50% [119]. The analysis of QoL in hospices indicated a number of significant predictors of poor outcome, such as socio-demographic characteristics (marital status, occupation, and education), and also psycho-physical phenomena, such as pain, anxiety, faith, and instrumental support [119]. Identification of factors improving QoL is of particular importance in the advanced stage of neoplastic disease.

Vitamin C can be considered as one of such agents, especially that the results of studies investigating the influence of high-dose IVC on QoL in cancer patients seem to be promising. In a multicenter, open-label, prospective study, Takahashi et al. [102] observed a significant improvement in the QoL assessed by the EORTC QLQ C-30 questionnaire after two and four weeks of high-dose IVC therapy in patients with newly diagnosed cancer. The better QoL resulted not only from the improved self-assessed global health status and functioning, but also from the reduction of fatigue, pain, insomnia, and constipation intensity [102]. Similarly, Vollbracht et al. [105] demonstrated that the administration of IVC with standard anti-cancer therapy in the first postoperative year of women with breast cancer resulted in significantly enhanced performance status and reduction of complaints induced by the disease and chemo-radiotherapy.

To answer the question of whether the high-dose IVC therapy is equally effective in patients with advanced-stage cancer, we identified a total of six studies including 105 patients: four non-randomized phase I or II studies (*n* = 103 participants) [27,28,29,120] and two case reports (*n* = 2 participants) [121,122]. All participants of analyzed studies had advanced malignancies with distant metastases or cancer disease refractory to standard anti-cancer therapy.

Unfortunately, the studies conducted among patients in the advanced stage of cancer did not give such unequivocally positive results. In a prospective uncontrolled study, Yeom et al. [120] noticed statistically significant improvement in quality of life assessed by the EORTC QLQ-C30 questionnaire after double intravenous administration of 10 g vitamin C with a 3-day interval followed by oral intake of 4 g vitamin C daily for a week. The study was performed in terminal cancer patients in whom anti-cancer therapy was no longer conducted. Improvement in QoL was observed in patients’ self-evaluation of their global health and quality of life (global health scale) as well as all aspects of functioning (function scale): in the physical, role, emotional, cognitive, and social field. Moreover, study participants observed statistically significant reduction of intensity of some symptoms, such as fatigue, nausea/vomiting, pain, sleep disturbance, and loss of appetite [120]. In a phase I clinical trial by Stephenson et al. [28], high-dose IVC was applied as a monotherapy in patients with advanced solid tumors refractory to standard therapy. The average values for the functioning and symptom scales of the EORTC QLQ-C30 remained constant for the first two weeks of therapy with a trend to improve for those few patients, who completed the questionnaire at the 3rd and 4th weeks (*n* = 7 and *n* = 2, respectively) [28]. Unfortunately, the authors of the study did not demonstrate statistical significance of those changes. Improved QoL, together with simultaneously reduced intensity of symptoms and fatigue, was reported also by Carr et al. [121,122] in patients with recurrent cancers.

On the other hand, Hoffer et al. [29] in a phase I dose-escalating trial of IVC in patients with advanced malignancy refractory to standard therapy, observed a significant deterioration in patients’ physical condition over the course of the study, assessed by the Functional Assessment of Cancer Therapy-General (FACT-G) questionnaire. Interestingly, the above observation was made only for patients who received IVC in a dose of 0.4 g/kg, while there was no deterioration of physical condition among patients who received higher doses of ascorbic acid. No changes in the social, emotional, and functional parameters of QoL were reported in any cohort of the study [29]. Similarly, Nielsen et al. [27] in a single-center phase II clinical trial in metastatic, castration-resistant prostate cancer patients found a significant deterioration in physical functioning with no trends towards improvement for the other QoL scales. What is more, 80% of study participants had an unchanged ECOG score at the 12th week of the study, while it improved in two patients and aggravated in another two compared to baseline [27].

The above-mentioned studies indicate that a favorable effect of high-dose IVC on QoL observed in certain advanced cancer patients comprises various elements. The improved patient’s performance status and reduction in the severity of symptoms seem to be most important. In the studies conducted so far, improved QoL has been observed simultaneously with the relief of pain, fatigue, lack of appetite, nausea/vomiting, and sleep disorders. Anti-oxidant and anti-inflammatory features and/or enzyme cofactor function of vitamin C may also be involved in QoL improvement [25]. Some researchers emphasize the positive impact of ascorbate on the central nervous system, mental skills, and contribution to enhanced vigor [120]. Effects of intravenous vitamin C on QoL in the context of performance status and severity of symptoms in cancer patients are presented in Table 1.

## 11. Does High-Dose IVC Affect the Cancer-Related Fatigue in Advanced-Stage Cancer Patients?

Cancer-related fatigue (CRF) is defined as persistent, subjective, and distressing sense of physical, emotional, and/or cognitive tiredness or exhaustion related to cancer or anti-cancer therapy, that is non-proportional to undertaken physical activity and interferes with patient’s usual functioning [124]. Cancer patients affected by CRF vary widely in their manner of expressing the problem, describing CRF as a feeling of exhaustion, lack of energy, loss of drive and personal interests, as well as impaired memory and concentration [125]. CRF involves a vicious circle of diminished physical performance, inactivity, avoidance of effort, absence of regeneration, helplessness, and depressed mood [125] and differs from other types of fatigue by its persistence, intensity, and the inability to be alleviated after resting or sleeping [124]. Prevalence of CRF as the most common symptom experienced by cancer patients is estimated to range from 60 to 90%, depending on patients sampling and application of methodology used [126,127]. Up to 40% of patients report fatigue at the time of cancer diagnosis and about 80–90% experience CRF during chemotherapy and/or radiotherapy [124]. What is more, it may persist for years after anti-cancer treatment has finished [128]. In the palliative care setting, CRF is described as a “clinically important” to “severe” problem by 48–75% patients [126], thus it is a main cause of disruption in all aspects of patients’ quality of life [128].

In the recent European Society for Medical Oncology (ESMO) recommendations for the diagnosis and treatment of cancer-related fatigue, IVC was not considered as a remedy that can reduce intensity of cancer-related fatigue [124]. However, the studies carried out so far revealed that IVC alone or in combination with other medications such as omega-3 polyunsaturated fatty acids (n-3 PUFA) reduces fatigue in healthy office workers [129] and postoperative fatigue among patients undergoing coronary artery bypass grafting (CABG) surgery as well [130]. Some studies conducted so far among cancer patients seem to be promising and worth mentioning.

To answer the question whether the high-dose IVC therapy is effective in relieving of CRF, we identified a total of eight studies including 144 patients with disseminated or refractory to anti-cancer therapy malignancies: four non-randomized phase I or II studies (*n* = 103 participants) [27,28,29,120], one controlled retrospective study (*n* = 39 participants), [123] and two case reports (*n* = 2 participants) [121,122]. Admittedly, we analyzed the effect of high-dose IVC on CRF in cancer patients undergoing chemotherapy. For this purpose, we identified two studies meeting these criteria, including 185 patients: one prospective study with 60 participants [102] and one controlled retrospective study conducted among 125 breast cancer patients [105].

Statistically significant reduction in CRF (*p* = 0.001) with concomitant improvement in all functioning scales (*p* < 0.05) (physical, role, emotional, cognitive, and social functioning) in the end-stage cancer patients receiving high-dose IVC as the only supportive treatment for one week was observed in the prospective study of Yeom et al. [120]. Similar observations were made by Stephenson et al. [28], however no data on statistical significance was provided in the study. Gunes-Bayir et al. [123], in a controlled retrospective study, observed improved performance status in 27% of patients with metastatic, radiotherapy-resistant malignancies after IVC therapy and in 7% of those undertaken chemotherapy, respectively. In the control group, receiving no intervention, performance status worsened during the study [123]. On the other hand, Nielsen et al. [27] found no change in subjective fatigue intensity in advanced prostate cancer patients, but observed a significant (*p* < 0.01) deterioration in physician functioning after 12 weeks of IVC administration. Details of the above mentioned studies are shown in Table 1.

In a prospective study by Takahashi et al. [102], patients with newly diagnosed cancer, with anti-cancer therapy administered in 57% cases, reported statistically lower fatigue intensity (*p* < 0.01) accompanied by improvement in physical (*p* < 0.05) as well as role, emotional, cognitive, and social functioning (*p* < 0.01) after 4 weeks of IVC therapy. The positive impact of high-dose IVC on patients’ performance status was also demonstrated by Vollbracht et al. [105] in the epidemiological retrospective study with 125 women with primary non-metastasized breast cancer, receiving a basic anti-tumor therapy with IVC at a dose of 7.5 g once a week for at least 4 weeks. Women who received IVC had a significantly better performance status during adjuvant therapy and the aftercare follow-up than those who did not receive IVC. During the 6th month of adjuvant treatment, the mean score of the Karnofsky index in a study group was significantly higher (*p* < 0.001) than in the control group (80% vs. 71%, respectively) and the mean score of the European Cooperative Oncology Group (ECOG) Performance Status scale was significantly lower (1.596 in a study group vs. 2.067 in the control group, *p* = 0.002), which proved the positive effect of vitamin C on patients’ functional status [105].

As it was shown in studies presented above, a positive effect on CRF can be more expressed in patients with basically better performance status and in patients with chemotherapy-related fatigue, whereas CRF in advanced-stage cancer patients refractory to standard therapy with features of disease progression may benefit less or not at all from IVC therapy. In the above mentioned studies, it was revealed that the duration of a study and the moment of patients’ observation can influence obtained results. The functional status assessed after 12 weeks of vitamin C treatment showed a deterioration which was not observed in studies of shorter duration (e.g., 4 weeks). The former observation probably results directly from the progression of already advanced cancer disease taking place after that time.

Although the etiology of CRF has not yet been clearly elucidated, several cytokines and other pro-inflammatory mediators (interleukin IL-6, IL-1, and neopterin) produced in response to cancer have been proposed to be associated with this phenomenon [124]. It was observed that cancer patients present with elevated inflammatory and angiogenesis promoting cytokines (e.g., M-CSF-R, Leptin, EGF, FGF-6, TNF-α, TNF-β, TARC, MCP-1, MCP-44, MIP, IL-4, IL-10, and TGF-β) compared to healthy controls [52]. Moreover, it has been shown that high-dose IVC (15–50 g up to three times a week) resulted in reduced CRP levels (in 76 ± 13% of study participants) and reduced concentration of pro-inflammatory cytokines (IL-1α, IL-1β, IL-2, IL-8, tumor necrosis factor TNF-α), chemokines (eotaxin, e-selectin, lymphotactin, MIP-1, MCP-1, TARC, SDF-1), and mitogens (EGF, Fit-3 ligand, IGF-1, IL-21R) in blood [52]. Thus, the anti-inflammatory properties of vitamin C may be responsible for the fatigue-relieving effect.

Enhanced oxidative stress in cancer patients mediated by neoplastic disease itself and chemotherapeutic agents can seriously affect non-targeted tissues, such as striated muscles, leading to their dysfunction, weakness, and fatigue [131,132]. Vitamin C is well known as a potent antioxidant possessing the ability to scavenge free radicals and reactive oxygen species, thus decreasing markers of oxidative stress in vivo [133]. High tissue levels of ascorbate may provide antioxidant protection from this oxidant-mediated toxicity observed in cancer patients [131]. As an essential micronutrient in the central nervous system (CNS), vitamin C serves important functions including antioxidant protection, peptide amidation, myelin formation, and synaptic potentiation [134]. It can have a beneficial impact on CNS through an increase in brain c-AMP levels and prevention of the formation of toxic oxidative forms of some neurotransmitters (such as epinephrine or norepinephrine) [120]. Moreover, acting as a cofactor in the synthesis of neurotransmitters, such as norepinephrine, dopamine, and serotonin, ascorbate participates in maintaining their proper concentrations in CNS [133]. All above mentioned mechanisms might be important in improving the performance status and physical functioning in patients with CRF.

On the other hand, a number of studies conducted so far have revealed high incidence of vitamin C deficiency among cancer patients. Vitamin C deficiency itself can result in such symptoms as fatigue and weakness. Vitamin C plays an important role in the production of energy in beta-oxidation processes; it is necessary for two dioxygenase enzymes involved in the biosynthesis of carnitine, acting as an essential cofactor in the transport of long-chain fatty acids into the mitochondria [131]. Therefore, the impaired carnitine metabolism (because of insufficient vitamin C supplementation or excessive use in states of pathology) can be responsible for weakness [108]. Therefore the improvement in functioning scales and reduction in intensity of fatigue reported by cancer patients after intravenous vitamin C supplementation might result from the improvement in plasma vitamin C saturation.

## 12. Potential Analgesic Properties of High-Dose Vitamin C in Cancer-Related Pain

Pain is one of the most common symptoms reported by cancer patients, which strongly deteriorates their quality of life and is associated with negative psychosocial responses [135]. Cancer pain can be related to primary tumor, disease progression, and metastasis as well as to anti-cancer therapy and its complications. Despite increased attention on assessment and management of pain in the last decade, its prevalence and intensity still remain substantial. Pain is reported by 66.4% of patients in advanced, metastatic, or terminal disease, 55% of cancer patients during anticancer therapy and 39% of subjects after anticancer treatment [135]. What is more, 38% of all cancer patients experience moderate to severe pain, which indicates the need for development and implementation of new interventions providing optimal management of cancer-related pain [135]. The nature of cancer-associated pain is usually complex, consists of nociceptive, neuropathic, and inflammatory components, thus requiring a complex approach and treatment with the use of analgesics with different mechanisms of action, such as opiate receptor agonists, acetaminophen, and/or non-steroidal anti-inflammatory drugs in combination with adjuvant drugs (co-analgesics). The term “adjuvant analgesic” or “co-analgesic” concerns any drug with a major clinical use other than pain that is helpful as an analgesic in certain circumstances, providing additional analgesia in specific types of pain (e.g., antiepileptics, gabapentin, and pregabalin, are indicated as first-line therapy for neuropathic pain). Taking into account the following biological properties of vitamin C, it may be one of nutraceuticals used in palliative care as “adjuvant analgesic”.

As it was mentioned above, cancer patients’ population in a large percentage is affected with vitamin C deficiency. It is worth mentioning that musculoskeletal pain is one of symptoms observed in serious vitamin C deficiency, scurvy [33]. It is presented mainly with arthralgia or myalgia, primarily due to bleeding into musculoskeletal tissues (muscles and joints) [33,136]. Data from epidemiological study conducted by Dionne et al. [137] among 4742 healthy participants aged ≥20 years revealed the association between suboptimal vitamin C plasma concentration and spinal pain occurrence. It proves that the pain reported by cancer patients, mainly the one referred to musculoskeletal system may be related both to the cancer itself and/or its treatment, but also in some cases, to vitamin C deficiency. Studies conducted so far among cancer patients have indicated that high-dose IVC in some circumstances might exert analgesic effects.

Cameron and Campbell [7] were the first who reported in 1974 a reduction in pain intensity in terminal cancer patients receiving intravenous ascorbic acid for a period of no longer than 10 days (details are shown in Table 2). In a subsequent prospective study conducted in the 2007 by Yeom et al. [120], a significant alleviation of pain was observed among 39 terminal cancer patients after IVC administration in monotherapy. Similar analgesic effect of IVC was observed in 60 patients with newly diagnosed cancer (56% on anti-cancer treatment) at the 4th week of IVC therapy (25–100 g weekly) [102]. Stephenson et al. [28], in a prospective study with 17 patients with advanced solid tumors refractory to standard therapy, observed a gradual reduction in pain intensity until its complete disappearance at the 4th week of IVC therapy, but no statistical analysis was performed in the study. Carr et al. [121,122] presented two case reports demonstrating improvement in a control of pain after IVC therapy. Details of above mentioned studies are shown in Table 2.

It is worth mentioning that in the above studies, pain was assessed by using a subjective scales (EORTC QLQ-C30 questionnaire, numerical, or verbal scales), therefore the results obtained may not be considered reliable. However, it should be emphasized that the perception of pain sensations and its intensity is always a patient’s subjective self-assessment and there are no known other objective methods of pain intensity assessment.

It remains uncertain, which of the biological properties of vitamin C is responsible for its potential analgesic effect. It was shown by Mikirova et al. [52,53] that high-dose IVC may reduce inflammation in cancer patients, and these anti-inflammatory properties of vitamin C may play a key role in certain clinical situations, such as cancer-induced bone pain. In the area of bony metastases, a wide range of pro-hyperalgesic mediators commonly associated with inflammation is released from the tumor and related immune cells, all of which are likely to contribute to cancer-induced bone pain. Prostaglandins, endothelins, bradykinin, tumor necrosis factor-α (TNF-α), and a range of growth factors such as transforming growth factor β (TGF-β) not only sensitize peripheral nociceptors to subsequent stimuli, but also have a direct impact on specific receptors on the primary sensory neurons. Several clinical studies conducted so far have revealed a significant reduction in bone pain in cancer patients with bone metastases after IVC treatment, thus confirming these assumptions. The majority of patients with partial or even complete resolution of pain in a study of Cameron and Campbel [7] were those with bone pain. In addition, Günes-Bayir et al. [123] and Kiziltan et al. [138], in retrospective studies, found about 50% reduction in pain intensity among patients with radiotherapy-resistant bone metastases after IVC administration.

Animal studies revealed that vitamin C is able to affect the pain modulating pathway in the spinal cord, thus inhibiting neuropathic pain [139]. Its analgesic properties might be related to participation in the synthesis of catecholamine neurotransmitters [136]. Vitamin C is a cofactor for the enzyme dopamine β-hydroxylase which converts dopamine into norepinephrine. It may be also involved in the synthesis of dopamine and serotonin [136,140]. Recent studies of animal models of neuropathic pain indicated that noradrenaline plays a special role in the central inhibition of neuropathic pain; additionally, this effect may be amplified by serotonin and dopamine [141]. Noradrenaline in the spinal cord directly inhibits neuropathic pain through α_2_-adrenergic receptors [141]. Moreover, increased noradrenaline concentration acts on the locus coeruleus and improves the function of the impaired descending noradrenergic inhibitory system [141], which is crucial in the central control of pain. In ascorbate-deficient laboratory animals, decreased norepinephrine concentrations were found [136]. No such correlation has been investigated in human studies, however it was revealed that vitamin C deficiency possibly increases the risk of postherpetic neuralgia (PHN). Plasma concentrations of vitamin C were significantly lower in patients with PHN than in healthy volunteers [142,143,144]. Moreover, ascorbate supplementation effectively restored plasma vitamin C concentrations with concomitant decrease in spontaneous pain related to PHN [142]. As postherpetic neuralgia is an example of neuropathic pain, an observed effect of ascorbic acid may additionally explain the validity of IVC supplementation as a co-analgesic in cancer patients, especially in states of vitamin C deficiency and in such a type of pain. Furthermore, animal models of neuropathic pain demonstrated that vitamin C can enhance gabapentin analgesic effect [145,146]. Ascorbic acid given alone was also able to produce a dose-dependent antinociceptive effect [146,147,148].

Another possible mechanism of vitamin C-related analgesia results from its potential role in the synthesis of amidated opioid peptides as a cofactor for enzyme peptidylglycine α-amidating mono-oxygenase (PAM) [136]. Many amidated neuropeptides have potent opioid agonist activity, therefore vitamin C can act as an opioid-sparing agent when used as adjuvant therapy in the management of chronic cancer-related pain [149]. It is supported by the results of the observational study by Cameron and Campbel [7] that vitamin C administration reduced requirement for opioid analgesics with no further need for opioids in some cases.

## 13. Safety of High-Dose Vitamin C Treatment in Advanced-Stage Cancer Patients

High-dose IVC is considered to have a relatively good safety profile providing that appropriate precautions are taken, although it also can cause serious side effects in some patients. Vitamin C in gram doses is contraindicated in patients with glucose-6-phosphate dehydrogenase (G6PD) deficiency due to risk of developing intravascular hemolysis [150,151]. For this reason, red blood cell G6PD screening is required before applying vitamin C therapy. However, even when administered orally, vitamin C might induce hemolysis in patients with paroxysmal nocturnal hemoglobinuria [152]. On the other hand, because the metabolic end-product of vitamin C is oxalate, an acute oxalate nephropathy has been observed, especially when gram doses of IVC were given as a prolonged treatment in patients with chronic renal disease [153,154]. It can also worsen iron overload in patients with hemochromatosis or those who receive repeated transfusions of concentrated red blood cells [23]. Unfortunately, there is no clear data on adverse effects and possible high-dose IVC-induced toxicities among patients with cancer cachexia and those presenting features of dehydration who constitute a high percentage of end-stage disease patients.

During IVC administration, patients frequently reported mild light-headedness and nausea which resolved after eating and drinking, probably resulting from the osmotic load [32]. Transient treatment-related vomiting, thirst, dry mouth and skin, increased urinary flow, diarrhea, and headache, alleviating within the same day as infusion, were most frequently reported [26,28,30,58]. Some patients experienced unpleasant fluttering sensation in the upper abdomen and chills during the IVC infusion and increased leg edema for a few days after each infusion [26]. Among other complaints, hypertension, insomnia, abnormal urine color, loss of appetite, fatigue, flu-like symptoms, facial flushing, and perspiration were reported [27,28,102] (details are shown in Table 3.). Moderate to severe laboratory abnormalities, such as hypernatremia, hypokalemia, hypercalcemia, and low hemoglobin count were also observed [27,28,58]. Single serious adverse events observed during vitamin C treatment, such as pulmonary embolism and pneumonia rather seem to be related to cancer progression and subsequent complications than result from IVC treatment.

## 14. Summary

Intravenously administered vitamin C is widely used by complementary and alternative medicine practitioners, most often due to infection, cancer, and fatigue [2]. Results obtained from in vitro studies demonstrated that millimolar ascorbate plasma concentrations, achievable only after intravenous vitamin C (IVC) administration, were cytotoxic to the fast-growing malignant cells and enhanced anti-cancer effect of chemotherapeutic agents. Although these results were very promising and consistent with data obtained from animal studies, where injections of high-dose vitamin C inhibited tumor growth and prolonged the survival of laboratory animals, both high-dose IVC sole treatment and IVC in combination with standard chemotherapy were found ineffective in human studies conducted in advanced-stage cancer patients.

High-dose IVC might be considered as a part of palliative care, since improved quality of life and better global health status were reported in some prospective clinical studies. However, its effects can be influenced by patient’s baseline general condition and the stage of the disease. Although assessment of quality of life is always subjective and a placebo effect cannot be excluded in that case, such treatment is worth considering, especially as improving the quality of life is a major focus of palliative care. It was also shown that a positive IVC effect on CRF can be more expressed in patients with basically good performance status and in patients with chemotherapy-related fatigue, whereas CRF in advanced-stage cancer patients refractory to standard therapy with features of disease progression may benefit less or not at all from IVC therapy. Moreover, it has been also shown that high-dose IVC can act as an analgesics especially in cancer-related bone pain, but further studies are required to better understand its analgesic properties and to confirm its effectiveness in placebo-controlled trials. The most common myths about effectiveness of high-dose IVC treatment with appropriate explanations are presented in Table 4.

High-dose vitamin C treatment seems to be safe in advanced cancer patients when appropriate precautions are taken with a risk of not serious side effects, such as transient headache, dizziness, nausea, flu-like symptoms, and abnormalities in the results of laboratory tests (anemia, hypernatremia, hypercalcemia, elevated liver, and kidney parameters).

Summarizing, high-dose intravenous vitamin C treatment can be considered as palliative treatment in advanced-cancer patients, however its effects can be influenced by many factors, such as the patient’s baseline general condition, comorbidities, pathogenesis of the reported symptoms (e.g., pain), and the stage of the disease which can determine the occurrence of serious side effects (acute renal failure, hypercalemia, anemia). Further studies are required to better understand the above described vitamin C properties and its effectiveness should be determined in high-quality controlled studies with appropriate comparators.

## Figures and Tables

**Table 1:** Effect of high-dose intravenous vitamin C (IVC) on quality of life, performance status, and symptom severity in cancer patients.

Type of Study	Patients	Intervention	Outcome	References
Prospective	39 terminal cancer patients	10 g IVC twice with a 3-day interval for one week, followed by oral intake of 4 g daily for one week	Improved QoL assessed by the EORTC QLQ-C30 questionnaire: - improved global health status * - improved all functional scales * (physical, role, emotional, cognitive, social) - reduced intensity of symptoms: fatigue *, nausea/vomiting *, appetite loss *, pain *, insomnia *, dyspnea, constipation, diarrhea	Yeom et al. [120]
Prospective	24 patients with advanced cancer and hematological malignancy refractory to standard therapy	IVC three times a week at fixed doses 0.4, 0.6, 0.9 and 1.5 g/kg for average 10 weeks	QoL assessed by the FACT-G questionnaire: - worsening of physical function in 0.4 g/kg cohort *: - no changes in the social, emotional, and functional parameters of QoL in 0.4 g/kg and other cohorts	Hoffer et al. [29]
Prospective	60 patients with cancer, anti-cancer therapy administered in 34 patients	4 weeks of IVC therapy, median single dose 50 g (range 25–100 g)	Improved QoL assessed by the EORTC QLQ-C30 questionnaire: - improved global health status * - improved all functional scales *: physical, role, emotional, cognitive, social - reduced intensity of symptoms: fatigue *, insomnia *, constipation *, pain *, appetite loss, dyspnea, diarrhea	Takahashi et al. [102]
Prospective	17 patients with advanced solid tumors refractory to standard therapy	IVC for 4 consecutive days a week for 4 weeks, starting at 30 g/m^2^, a dose was increased by 20 g/m^2^ until a maximum tolerated dose (110 g/m^2^)	Improved QoL assessed by the EORTC QLQ-C30 questionnaire: - improved global health status - improved functional scales (physical, role, emotional, cognitive, social) - reduced intensity of symptoms: fatigue, nausea/vomiting, pain, dyspnea, insomnia, appetite loss, diarrhea	Stephenson et al. [28]
Prospective	23 patients with metastatic castration-resistant prostate cancer	IVC once weekly: 5 g in 1st week, 30 g in 2nd week, 60 g in 3–12 weeks	QoL assessed by the EORTC QLQ-C30 questionnaire: - no changes in global health status - worsening of physical * and role functioning with no changes in other functional scales (emotional, cognitive, social) - no changes in symptoms intensity QoL assessed by the QLQ-PR25 questionnaire: - no changes in functional scales - no changes in symptoms intensity	Nielsen et al. [27]
Controlled retrospective	125 patients with breast cancer on anti-tumor therapy (study group *n* = 53 ; control group *n* = 72)	Study group: IVC 7.5 g once a week during adjuvant therapies for a minimum 4 weeks Controls: no IVC during adjuvant therapies	Improved performance status assessed by the Karnofsky index and the ECOG scale during the 6 months of study * and the next 6 months of aftercare * Reduced intensity of complains (study group vs. controls): - during the 6 months of study: fatigue *, sleep disorders *, loss of appetite *, depression * - during the next 6 months of aftercare: loss of appetite *, dizziness *, nausea *, fatigue *, sleep disorders *, hemorrhagic diathesis *	Vollbracht et al. [105]
Controlled retrospective	39 patients with bone metastases, radiotherapy-resistant (*n* = 15 on chemotherapy; *n* = 15 on IVC therapy; *n* = 9 controls)	IVC group: 2.5 g IVC during pain	Improved performance status assessed by the ECOG scale in 27% of IVC group and 7% of the chemotherapy group, while worsened in the control group	Günes-Bayir et al. [123]
Case study	A 45-year old female with recurrent breast cancer	IVC 50 g twice a week for 4 weeks	Improved QoL assessed by the EORTC QLQ-C30 questionnaire: - improved global health status - improved functional scales: physical, role, emotional, cognitive, social - reduced intensity of symptoms: fatigue, nausea/vomiting, pain, insomnia, loss of appetite Fatigue assessed by the MFSI-SF questionnaire: - reduced general, physical, emotional, and mental fatigue - enhanced vigor	Carr et al. [121]
Case study	A 81-year-old male with recurrent pulmonary angiosarcoma	IVC 30 g daily for 1 week	Improved QoL assessed by the EORTC QLQ-C30 questionnaire: - improved global health status - improved functional scales (physical, role, emotional, cognitive, social) - reduced intensity of symptoms: fatigue, nausea/vomiting, pain, insomnia, loss of appetite Fatigue assessed by the MFSI-SF questionnaire: - reduced general, physical, emotional, and mental fatigue	Carr et al. [122]

Quality of life was assessed using the EORTC QLQ-C30 questionnaire unless otherwise indicated. * *p* < 0.05 compared to values before IVC therapy (if shown in the study). EORTC QLQ-C30—The European Organization for Research and Treatment of Cancer Quality-of-Life Core-30; FACT-G—Functional Assessment of Cancer Therapy-General; QLQ-PR25—Quality of Life Questionnaire–Prostate Module 25; MFSI-SF—Multidimensional Fatigue Syndrome Inventory-Short Form.

**Table 2:** Effect of high-dose intravenous vitamin C (IVC) on pain severity in cancer patients.

Study Type	Characteristics of Study Participants	Intervention	Outcome	References
Prospective	39 terminal cancer patients	10 g IVC twice with a 3-day interval in the first week, followed by oral intake of 4 g daily for one week	Reduced intensity of pain after IVC treatment	Yeom et al. [120]
Prospective	60 patients with newly diagnosed cancer, anti-cancer therapy administered in 34 patients	4 weeks of IVC therapy, median single dose 50 g (range 25–100 g)	Reduced intensity of pain after IVC treatment	Takahashi et al. [102]
Prospective	17 patients with advanced solid tumors refractory to standard therapy	IVC for 4 consecutive days a week for 4 weeks, starting at 30 g/m^2^, increased until a maximum tolerated dose (110 g/m^2^)	Reduced intensity of pain during IVC with complete pain relief after 4 weeks of IVC therapy	Stephenson et al. [28]
Controlled retrospective	39 patients with bone metastases, radiotherapy-resistant (*n* = 15 on chemotherapy; *n* = 15 on IVC therapy; *n* = 9 controls)	IVC group: 2.5 g IVC during pain	Median 50% reduction in pain intensity in IVC group	Günes-Bayir et al. [123]
Uncontrolled retrospective	11 cancer patients with bone metastases, unresponsive to standard cancer treatment	IVC 2.5 g once weekly for 3–10 weeks	Mean 55% reduction in pain intensity	Kiziltan et al. [138]
Case study	A female aged 53 with breast carcinoma with visceral and skeletal metastases, after mastectomy, radiotherapy, and hormonotherapy, terminal state with severe pain requiring opioids	5 g IVC for 7 days, followed by 8 g daily for 70 days orally (total 595 g)	Complete relief from bone pain Reduced need for opioids	Cameron and Campbell [7]
	A male aged 44 with poorly differentiated transitional cell cancer of bladder with bone metastases, intense pain inadequately controlled by opioids	10 g IVC for 10 days, followed by 10 g daily for 24 days orally (total 340 g)	Complete relief from bone pain No further need for opioids	Cameron and Campbell [7]
	A female 49 with disseminated carcinoma of unknown origin, innumerable osteolytic bone metastases, severe bone pain	10 g IVC for 7 days, followed by 10 g daily for 27 days orally (total 340 g)	Complete relief from bone pain	Cameron and Campbell [7]
	A male aged 49 with large malignant tumor of right temporal lobe, unknown histology, intolerable headache	10 g IVC for 11 days, followed by 10 g daily for 2 days orally (total 360 g)	Significant relief from headache	Cameron and Campbell [7]
Case study	A female aged 45 with recurrent breast cancer	50 g IVC twice a week for 4 weeks	Reduction in pain intensity	Carr et al. [121]
Case study	A male aged 81 with recurrent pulmonary angiosarcoma	30 g IVC daily for 1 week	Reduction in pain intensity	Carr et al. [122]

**Table 3:** Adverse effects of high-dose intravenous vitamin C (IVC) in patients with advanced cancer observed in prospective studies.

Patients	Intervention	Adverse Effects	References
24 terminal cancer patients	150–710 mg/kg/day IVC for up to 8 weeks	Frequently reported: nausea, edema, dry mouth or skin Grade 3 adverse events: kidney stone (*n* = 1), hypokalemia (*n* = 1)	Riordan et al. [58]
24 patients with advanced metastatic solid tumor or hematological malignancy refractory to standard therapy	IVC 3 times a week at fixed doses 0.4, 0.6, 0.9 and 1.5 g/kg for average 10 weeks	Mild subjective symptoms: nausea (*n* = 3), diarrhea (*n* = 2), headache (*n* = 2), dizziness (*n* = 2), fatigue (*n* = 2), facial flushing (*n* = 2), abdominal cramps (*n* = 1), vomiting (*n* = 1), perspiration (*n* = 1)	Hoffer et al. [29]
14 patients with metastatic stage IV pancreatic cancer	IVC 50–100 g/d three times a week for 8 weeks along with standard treatment of gemcitabine and erlotinib	Frequently reported: dizziness, nausea Grade 1–2 adverse events: low platelet count (*n* = 8), low hemoglobin count (*n* = 1), low neutrophil count (*n* = 1), hyperglycemia (*n* = 1), gastrointestinal discomfort (*n* = 1), conjunctival infection (*n* = 1), ascites (*n* = 1) Grade 3–4 adverse events: internal bleeding (*n* = 1), pulmonary embolism (*n* = 2), hospitalization due to anemia and UTI (*n* = 1), ileus (*n* = 1) Grade 5 adverse events: death from disease progression (*n* = 1)	Monti et al. [32]
11 patients with advanced pancreatic cancer	IVC 15–125 g/d twice a week for 8 weeks along with standard treatment (gemcitabine)	Frequently reported grade 1–2 adverse events: nausea (*n* = 6), diarrhea (*n* = 4), dry mouth (*n* = 4) Grade 3–4 adverse effects: elevated plasma GGT (*n* = 2), hypokalemia (*n* = 1), leukopenia (*n* = 1), lymphopenia (*n* = 1), neutropenia (*n* = 2), thrombocytopenia (*n* = 1)	Welsh et al. [30]
17 patients with advanced solid tumors refractory to standard therapy	IVC for 4 consecutive days a week for 4 weeks, starting at 30 g/m^2^, doses increased by 20 g/m^2^ until a maximum tolerated dose (110 g/m^2^)	Grade 1–2 adverse events: HT (*n* = 4), hypernatremia (*n* = 2), hypoalbuminemia (*n* = 1), hypokalemia (*n* = 1), hyperglycemia (*n* = 1), hypercalcemia (*n* = 1), increased creatinine (*n* = 1), elevated plasma LDH (*n* = 1), proteinuria (*n* = 1), bacteremia (*n* = 1), granular casts (*n* = 1), lower back pain (*n* = 1), tumor fever (*n* = 1), pedal edema (*n* = 1), headache (*n* = 1), peripheral neuropathy (*n* = 1) Grade 3–4 adverse events: hypokalemia (*n* = 2), hypernatremia (*n* = 2), anemia (*n* = 2), headache (*n* = 1)	Stephenson et al. [28]
14 patients with advanced cancer	IVC 1.5 g/kg body weight 2 or 3 times a week in combination with chemotherapy	Frequently reported mild adverse events: thirst, increased urinary flow Others: nausea and occasional vomiting (*n* = 1), thirst and unpleasant sensation in the upper abdomen (*n* = 1), chills, thirst, headache, leg edema (*n* = 1)	Hoffer et al. [26]
23 patients with metastatic castration-resistant prostate cancer	IVC once weekly: 5 g in 1st week, 30 g in 2nd week, 60 g in 3–12 weeks	Grade 1–2 adverse events: anemia (*n* = 7), HT (*n* = 5), UTI (*n* = 4), elevation of plasma aminotransferase (*n* = 3), ↓GFR (*n* = 3), flu-like symptoms (*n* = 3), limb pain (*n* = 3), musculoskeletal lesion (*n* = 2), shortness of breath (*n* = 2), pneumonia (*n* = 1), diarrhea (*n* = 1), dry eyes (*n* = 1), osteoporotic fracture (*n* = 1), pre-syncope (*n* = 1), leukemia (*n* = 1), AF (*n* = 1), elevated plasma bilirubin (*n* = 1), hydronephrosis (*n* = 1), hypercalcemia (*n* = 1), hyponatremia (*n* = 1) Grade 3–4 adverse event: pulmonary embolism (*n* = 1), pneumonia (*n* = 1)	Nielsen et al. [27]

Evaluation of all adverse events are based on the National Cancer Institute Common Terminology Criteria for Adverse Events 3.0 (if available in the study). UTI—urinary tract infection; GGT—gamma-glutamyl transferase; HT—hypertension; LDH—lactate dehydrogenase; AF—atrial fibrillation.

**Table 4:** Facts and myths about high-dose intravenous vitamin C (IVC) effectiveness in advanced-stage cancer patients.

Myths	Facts
High-dose IVC is a potent anticancer treatment because of its proven effectiveness in preclinical in vitro and animal studies	No consistent evidence for anti-cancer efficacy of high dose IVC therapy in patients with advanced-stage cancer in terms of objective tumor-related response or improved survival outcomes.
High-dose IVC treatment enhances effectiveness of conventional anticancer therapy	No reliable evidence for increased effectiveness of combined IVC/conventional therapy compared to standard chemotherapy.
High-dose IVC treatment reduces chemotherapy-induced toxicity	No reliable evidence for decreased chemotherapy-induced toxicity after combined IVC/conventional therapy compared to standard chemotherapy.
High-dose IVC reduces fatigue in cancer patients	Positive effect of high-dose IVC on cancer-related fatigue comprises various factors and it can be more expressed in patients with basically better performance status and in those with chemotherapy-related fatigue.
